# Interferon-induced protein IFIT4 is associated with systemic lupus erythematosus and promotes differentiation of monocytes into dendritic cell-like cells

**DOI:** 10.1186/ar2475

**Published:** 2008-08-15

**Authors:** Xiangyang Huang, Nan Shen, Chunde Bao, Yueying Gu, Li Wu, Shunle Chen

**Affiliations:** 1 Shanghai Institute of Rheumatology, Renji Hospital, Shanghai Jiao Tong University School of Medicine, Shan Dong Middle Road, Shanghai 200001, PR China; 2 Molecular Rheumatology Laboratory, Institute of Health Sciences, Shanghai Institutes for Biological Sciences, Chinese Academy of Sciences and Shanghai Jiao Tong University School of Medicine, Chong Qing South Road, Shanghai 200025, PR China; 3 Immunology Division, The Walter and Eliza Hall Institute of Medical Research, Royal Parade, Parkville, Victoria 3050, Australia

## Abstract

**Introduction:**

Using oligonucleotide microarray, many IFN-inducible genes have been found to be highly expressed in peripheral blood mononuclear cells (PBMCs) from most patients with systemic lupus erythematosus (SLE). Among these IFN-inducible genes, IFN-induced protein with tetratricopeptide repeats 4 (IFIT4) is a novel gene whose function is unknown.

**Methods:**

In this study we examined the role played by IFIT4 in monocyte differentiation and the correlation between IFIT4 expression and the clinical manifestation of SLE. To this end, we used plasmid transfection, flow cytometry, mixed leucocyte responses, ELISA, quantitative RT-PCR and Western blotting.

**Results:**

We found that both IFIT4 mRNA and protein expression levels were significantly higher in PBMCs and monocytes from SLE patients than in those from healthy control individuals. IFIT4 expression was positively correlated with antinuclear antibodies, anti-double-stranded DNA, and anti-Sm auto-immune antibodies in SLE. Patients with SLE exhibiting higher expression of IFIT4 had a higher prevalence of leucopenia, thrombocytopenia and C3/C4 decrease. IFIT4 protein was localized exclusively to the cytoplasm, and it was significantly upregulated by IFN-α in normal PBMCs. To determine the role played by IFIT4 in monocyte differentiation, the monocytic cell line THP-1 was transfected with pEGFP-IFIT4 expression plasmid and stimulated with granulocyte-macrophage colony-stimulating factor/IL-4 to generate IFIT4-primed dendritic cell-like cells (DCLCs). IFIT4-primed DCLCs acquired morphological characteristics of dendritic cells more quickly, with greater resemblance to dendritic cells, as compared with DCLCs primed with pEGFP-C1 control plasmid trasfection. Furthermore, they exhibited higher expressions of CD40, CD86, CD80, HLA-DR and CD83, along with lower expression of CD14; increased IL-12 secretion; and an increased ability to stimulate T-cell proliferation. In addition, IFIT4-primed DCLCs enhanced IFN-γ secretion (about 2.4-fold) by T cells compared with controls.

**Conclusion:**

Our findings suggest that IFIT4 might play roles in promoting monocyte differentiation into DCLCs and in directing DCLCs to modulate T-helper-1 cell differentiation; these actions might contribute to the autoimmunity and pathogenesis of SLE.

## Introduction

Systemic lupus erythematosus (SLE) is a chronic autoimmune disease with multiple organ involvement, in which autoantibodies induce tissue damage. IFN-α/β [1,2] and IFN-inducible genes (IFIGs) [3-5] are believed to play a major role in SLE.

IFN-α is a causative agent in the pathogenesis of SLE [6-8]. Elevated levels of IFN-α were detected in the sera of lupus patients [9], and IFN-α levels in sera correlate with disease severity and the generation of autoantibodies [10-13]. Patients treated with IFN-α occasionally develop antinuclear antibodies (ANAs), anti-double-stranded DNA antibodies (anti-dsDNAs) and autoimmune disorders [1,14-17] similar to those characteristic of SLE, but the mechanism by which IFN-α expression is associated with the generation of autoantibodies *in vivo *requires clarification [18].

Notably, IFN-α/β was found to upregulate major histocompatibility complex expression and to induce differentiation of monocytes into dendritic cells (DCs) [19-26], antigen-presenting cells that induce and regulate immune responses. An increased number of circulating plasma cells and the presence of autoreactive T and B cells in the sera of patients with SLE suggest that the disease might be driven by alterations in DCs. Moreover, Blanco and coworkers [2] reported that IFN-α in the sera of SLE patients can induce normal monocytes to differentiate into DCs. Thus, IFN-α may enhance autoimmune responses in SLE by inducing DCs [27]. However, the mechanism by which IFN-α induces monocytes to differentiate into DCs has not been elucidated.

Recently, a gene expression study using an oligonucleotide microarray showed that many IFIGs are highly expressed in the peripheral blood mononuclear cells (PBMCs) of most SLE patients [3,5,28-32], and IFIGs correlated with the production of autoantibodies and the clinical manifestations of SLE [3,4,18,30,31]. The IFIGs were thought to be responsible for the immunomodulatory properties of IFN, such as monocyte differentiation and anti-proliferation. For example, Ifi204 favours macrophage differentiation in myeloid progenitor cells [33].

Among the IFIGs, IFN-induced protein with tetratricopeptide repeats 4 (IFIT4) is a novel gene whose function was unknown until recently, when it was shown to be a key mediator of antiproliferative activity by enhancing the p21 and p27 proteins [34,35]. Induction of IFIT4 transcription by IFN-α depends upon the sequential activation of protein kinase Cδ, c-Jun amino-terminal kinase, and STAT1 (signal transducer and activator of transcription 1) [36]. Because IFIT4 can be induced by IFN-α [36], which is involved in monocyte differentiation [2,19,20,27,37], we were interested in testing whether IFIT4 was responsible for the effect of IFN-α on differentiation of monocytes into DCs [38].

In the present study we found that increased expression of IFIT4 in the PBMCs of patients with SLE positively correlated with the presence of autoantibodies (ANA, anti-dsDNA and anti-Sm), leucocytopenia and hypocomplementaemia. Compared with DC-like cells (DCLCs) primed with pEGFP-C1 transfection, IFIT4-primed DCLCs exhibited higher expression of CD40, CD80, CD86 and HLA-DR; lower expression of CD14; enhanced IL-12 secretion; and increased ability to stimulate T-cell proliferation. This indicates that IFIT4 may play a role in promoting monocyte differentiation into DCs. Moreover, IFIT4-primed DCLCs induced a differentiation bias of CD4^+ ^T cells into T-helper-1 (Th1) cells. These findings suggest that IFIT4 might play a role in the pathogenesis of SLE by promoting monocyte differentiation into DCs.

## Materials and methods

### Patients

A total of 108 patients with SLE and 46 normal healthy donors were recruited from the Department of Rheumatology, RenJi Hospital, which is affiliated with Shanghai Jiao Tong University School of Medicine (Table 1). All SLE patients fulfilled the diagnostic criteria of the American College of Rheumatology. All of study participants signed a patient material and informed consent form, approved by Renji Hospital Institutional Review Board.

**Table 1:** Demographic characteristics of the study subjects

Characteristic	SLE patients	Healthy donors	*P*
Participants (*n*)	108	46	-
Age (years)	32.52 ± 11.97	32.5 ± 14.32	0.83
Sex (*n*; female/male)	96/12	40/6	0.62
Onset age (years)	27.14 ± 10.0	NA	-
Disease duration (years)	5.4 ± 4.13	NA	-
SLEDAI-2K score (mean [range])	6 (3 to 8)	NA	-
Mean ESR (mm/hour)	61.58 ± 24.03	NA	-
Medical therapy (*n*)			
Prednisone >60 mg/day	11	NA	-
Prednisone 30 to 60 mg/day	44	NA	-
Prednisone 0 mg/day	6	NA	-
CTX	35	NA	-

FIT4 relative expression	37.84 ± 3.52	10.58 ± 2.64	0.00

All patients were of Chinese ethnicity. Unless stated otherwise, values are expressed as mean ± standard deviation. CTX, cyclophosphamide; ESR, erythrocyte sedimentation rate; IFIT4, interferon-induced protein with tetratricopeptide repeats 4; NA, not applicable; SLE, systemic lupus erythematosus; SLEDAI-2K, SLE Disease Activity Index 2,000.

### Samples and clinical index

Human PBMCs were isolated by Ficolle-Hypaque density gradient separation. IFIT4 mRNA levels were detected by quantitative RT-PCR in total RNA, isolated from the PBMCs of 108 SLE patients and 46 healthy donors with Trizol reagent. IFIT4 protein expression levels were determined by Western blotting in protein samples from the PBMCs of 24 patients with SLE and 24 healthy control individuals.

### Isolation of monocytes and T cells

Blood monocytes from three patients with SLE and three healthy control individuals were isolated using an immuno-magnetic bead method (Miltenyi Biotec, Bergisch Gladbach, Germany) and were used to examine the expression of IFIT4 protein via Western blotting. CD4^+ ^lymphocytes were purified from PBMCs via an indirect magnetic labeling system, namely the CD4 T Cell Isolation Kit (Miltenyi Biotec, Bergisch Gladbach, Germany). Purities were generally in excess of 95%. Freshly isolated T lymphocytes were used to measure mixed leucocyte responses (MLRs).

### Quantitative real-time RT-PCR for mRNA

For quantitative analysis of gene expression, total RNA was isolated using a Trizol reagent kit (Invitrogen, Carlsbad, CA, USA). cDNA was synthesized and fluorescence real-time RT-PCR was performed by using SuperScript™ III Platinum^® ^SYBR^® ^Green Two-Step qRT-PCR Kits (Invitrogen, Carlsbad, CA, USA) via the ABI PRISM 7900 system (Perkin-Elmer, Foster City, CA, USA). The following primers were used: IFIT4 forward: 5'-AACTACGCCTGGGTCTACTATCACTT-3'; IFIT4 reverse: 5'-GCCCTTTCATTTCTTCCACAC-3'; GAPDH forward: 5'-GAAGGTGAAGGTCGGAGTC-3'; GAPDH reverse: 5'-GAAGATGGTGATGGGATTTC-3'. All data were analyzed using ABI PRISM SDS 2.0 software (Applied Biosystems, Foster, CA, USA). Using the ΔCt method, GAPDH was co-amplified to normalize the amount of RNA [39].

### Western blotting

Western blotting was performed as described previously [34,39]. Protein from PBMCs and monocytes were harvested and transferred to polyvinylidene fluoride membranes. The membranes were incubated with monoclonal anti-human IFIT4 antibody (BD Clontech, Palo Alto, CA, USA) followed by horseradish peroxidase-linked secondary antibodies (Cell Signaling, Beverly, MA, USA). Equal protein loading for Western blots was confirmed by immunoblotting for β-actin.

### Plasmid construction and transient transfection

The cDNA fragment encoding IFIT4 was amplified by PCR from a plasmid template (ORFEXPRESS™ Gateway^® ^ORF Shuttle Clones; GeneCopeia, Frederick, MD, USA), into which the full-length human IFIT4 cDNA was inserted. PCR products were digested by *Hind*III restriction endonuclease and cloned into the pEGFP-C1 vector, generating plasmid pEGFP-IFIT4. An endotoxin-free kit (EndoFree Plasmid isolation Kit, Qiagen, Chatsworth, CA, USA) was used to purify the vectors. pEGFP-C1 was used as a negative control (BD Clontech, Palo Alto, CA, USA). For transient transfections, the plasmids mentioned above were transfected into THP-1 cells using Lipofectamine 2000 (Invitrogen, USA) or Cell Line Nucleofector Kits V (Amaxa Biosystems, Cologne, Germany).

### Confocal microscopy

To observe the subcellular location of IFIT4, THP-1 cells were transfected with pEGFP-IFIT4 or pEGFP-C1 empty vector. Forty eight hours later, cells were stimulated with 3000 μ/ml IFN-α for 3 days. The cells were examined under a confocal microscope (Leica TCS SP2, Leica Microsystems, Heidelberg, Germany), using DAPI for nuclear staining.

### Cell culture and generation of IFIT4-primed dendritic cell-like cells from THP-1 cells

The human monocytic cell line THP-1 (ATCC, Manassas, VA, USA) [40] was cultured in RPMI 1640 (Hyclone, Logan, UT, USA) supplemented with 10% foetal bovine serum and 2 mmol/l L-glutamine at 37°C in 5% carbon dioxide. The following human recombinant cytokines were used as stimulators: recombinant human granulocyte-macrophage colony-stimulating factor (GM-CSF; 50 ng/ml), IL-4 (20 ng/ml; R&D Systems, Minneapolis, MN, USA), and lipopolysaccharide (LPS; 100 ng/ml, *Escherichia coli *0111:B:4; Sigma, St. Louis, MO, USA).

To generate DC-like cells (DCLCs), THP-1 cells were treated with GM-CSF and IL-4 (GM-CSF/IL-4) [40-44] at the indicated concentrations for up to 6 to 12 days. To generate IFIT4-primed DCLCs, THP-1 cells were transfected with pEGFP-IFIT4; 36 hours later the cells were stimulated with GM-CSF/IL-4 for up to 5 to 7 days. The DCLCs derived from THP-1 transfected with pEGFP-C1 were designated controls. Mature and active DCLCs were generated from THP-1 cells that were treated with GM-CSF/IL-4 for 6 to 12 days and further cultured with LPS for an additional 2 days. Cultures were fed every 2 or 3 days by removing half of the medium and adding fresh medium with full doses of cytokines.

### Morphological examination

To assess the effect of IFIT4 on monocyte differentiation, the morphology of DCLCs primed by transfection with pEGFP-C1 or pEGFP-IFIT4 was examined every day using an Olympus IX-70 inverted microscope

### Monoclonal antibodies and flow cytometry

Purified mouse IgG_1_, κ isotype control (Catalogue no: 400101); PE mouse IgG_2b_, κ isotype control, PE anti-human CD86 (Catalogue no: 305406); CD83 (Catalogue no: 305308); HLA-DR (Catalogue no: 307606); CD1a (Catalogue no: 300106); and FITC anti-human CD14 (Catalogue no: 325604) were obtained from Biolegend (San Diego, CA, USA). FITC anti-human CD40 (Catalogue no: FAB617F) and PE anti-human CD80 (Catalogue no: FAB140P) were from R&D Systems, Minneapolis, MN, USA.

DCLCs were incubated with saturating concentrations of fluorochrome-conjugated monoclonal antibodies at 4°C for 30 minutes and then washed twice in phosphate-buffered saline containing 2% foetal bovine serum and fixed in 1% paraformaldehyde. Cells were analyzed with a FACSort (BD Biosciences, Mountain View, CA, USA). Appropriate fluorochrome or isotype control monoclonal antibodies of each antibody species were used as negative controls.

### Mixed leucocyte responses

Mature and active DCLCs, generated as described above, were harvested and γ-irradiated (3,000 rads) followed by incubation with 5 × 10^4 ^allogeneic CD4^+ ^T cells/well, at ratios of DCLCs to T cells of 1:10, 1:20 and 1:40. Three days later, [^3^H]thymidine was added (0.5 μCi/well) and the cells were incubated for another 18 hours. The cells were harvested and the incorporated radioactivity measured using a β counter (model LS3801; Beckman Coulter, Brea, CA, USA). Responses were reported as the mean of triplicate samples (mean [counts/minute cpm] ± standard deviation).

### Enzyme-linked immunosorbent assay

Mature and active DCLCs were generated as described above. Total IL-12 (p40 and p70) secreted by DCLCs primed with pEGFP-IFIT4 or pEGFP-C1 transfection was measured with the BIOSOURCE IL-12+p40 ELISA kit (Biosource Europe S.A., Nivelles, Belgium) [45].

To examine the effect of IFIT4-primed DCLCs on T-cell polarization, T cells (2 × 10^5^/well) were plated and cultured with γ-irradiated IFIT4-primed DCLCs at a ratio of DCLCs to T cells of 1:10 for 6 days. 10 ng/ml of phorbol 12-myristate 13-acetate (PMA) was added and the cells cultured for 1 more day. The supernatants were harvested to measure the concentrations of IL-4 and IFN-γ by using the Human IL-4/IFN-γ ELISA Kit (Biosource Europe S.A.).

### Statistical analysis

Two group comparisons of gene expression were assessed using unpaired *t*-test, or the nonparametric Mann-Whitney test when the data did not have a normal distribution [3,18,46,47]. Results are presented as the mean ± standard deviation unless specified otherwise. Correlations of IFIT4 mRNA expression levels with titre of auto-antibodies and SLE Disease Activity Index (SLEDAI) scores were determined using Spearman's rank correlation coefficient [47].

## Results

### Increased expression of IFIT4 mRNA and protein in PBMCs and monocytes from SLE patients

**Figure 1:**
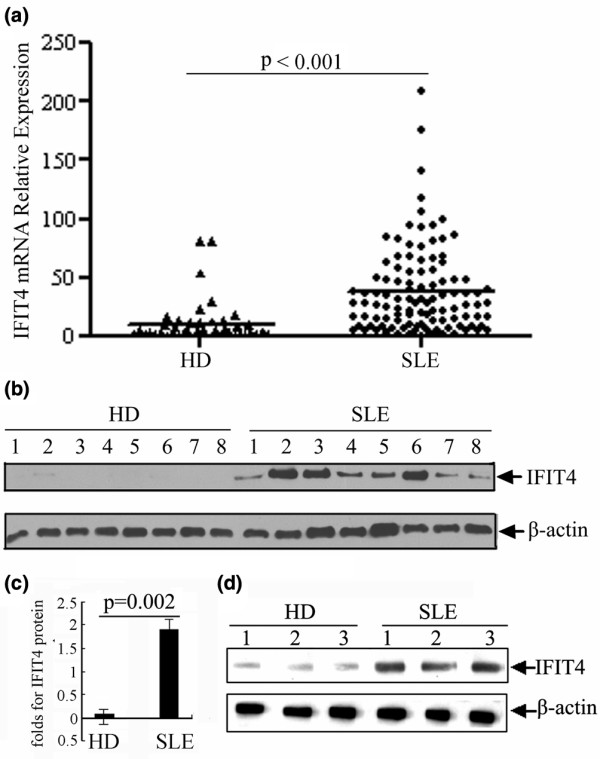
Expression levels of IFIT4 in patients with SLE and healthy control individuals. **(a) **Total RNA from the peripheral blood mononuclear cells (PBMCs) of 108 systemic lupus erythematosus (SLE) patients and 46 healthy donor (HDs) was isolated, and the relative expression level of IFIT4 mRNA was determined by real-time quantitative RT-PCR. Glyceraldehyde-3-phosphate dehydrogenase (GAPDH) was co-amplified as an internal control to normalize the amount of IFIT4 mRNA. All experiments were repeated three times with similar results. Horizontal lines indicate the mean (*P *< 0.001, Mann-Whitney test). **(b,c) **Total protein was extracted from the PBMCs of 24 SLE patients and 24 HD control individuals. IFIT4 protein expression levels were detected using Western blotting. β-Actin was used as a protein loading control. A set of random data from eight SLE patients and eight HD controls is presented (*P *= 0.002, Mann-Whitney test). **(d) **Monocytes from three SLE patients and three HDs were isolated using magnetic beads. The IFIT4 protein expression level in these monocytes was determined by Western blotting. IFIT4, interferon-induced protein with tetratricopeptide repeats 4.

A total of 108 SLE patients and 46 healthy donor individuals were matched for both age and sex (Table 1). The results of real-time quantitative RT-PCR revealed that the means of IFIT4 relative mRNA levels were 37.84 ± 3.52 in PBMCs from SLE patients and 10.58 ± 2.64 in those from healthy control individuals, and the difference was statistically significant (*P *< 0.001; Figure 1a). IFIT4 protein levels, examined using Western blotting, were significantly increased in PBMCs from 24 SLE patients than in those from the 24 healthy control individuals (*P *< 0.05; Figure 1b,c). In addition, IFIT4 protein levels in the monocytes of SLE patients were significantly increased compared with those in control individuals (*P *< 0.05; Figure 1d).

### Increased expression of IFIT4 is associated with autoantibodies in SLE

**Figure 2:**
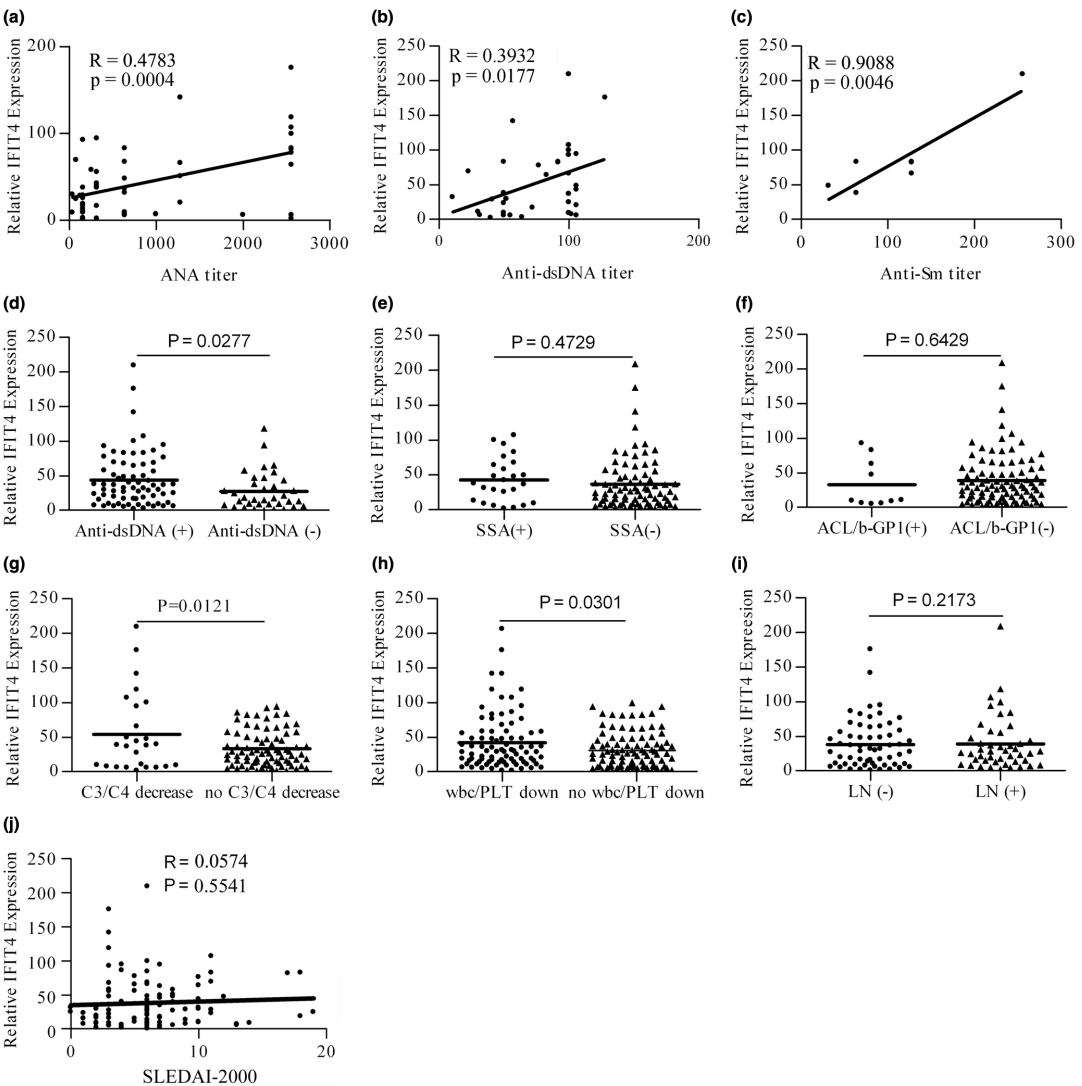
Correlation analysis between IFIT4 expression and clinical assessments in SLE patients. Total RNA from the peripheral blood mononuclear cells (PBMCs) of 108 systemic lupus erythematosus (SLE) patients and 46 healthy donors (HDs) was isolated, and the relative expression levels of IFIT4 mRNA were determined using real-time quantitative RT-PCR. Glyceraldehyde-3-phosphate dehydrogenase (GAPDH) was coamplified as an internal control to normalize the amount of RNA. In the total SLE population, the relative expression of IFIT4 was plotted against the following: **(a) **the titre of antinuclear antibody (ANA) in a group of 108 SLE patients; **(b) **the titre of anti-double-stranded DNA antibody (anti-dsDNA) in 36 SLE patients; and **(c) **the titre of anti-Smith antibody (anti-Sm) in 7 SLE patients. Spearman's correlation test was used to analyze these data. In the SLE population as a whole, the relative expression of IFIT4 was determined using real-time quantitative RT-PCR in patients who were positive or negative for the following: **(d) **anti-dsDNA antibody; **(e) **anti-SSA antibody; **(f) **anti-cardiolipid antibody or β-GP1 (ACL/b-GP1); **(g) **hypocomplementaemia (C3/C4 decrease); **(h) **haematocytopenia (wbc/PLT decrease); and **(i) **lupus nephritis. Mann-Whitney test was used to analyze these data. **(j) **The relative expression of IFIT4 was plotted against Systemic Lupus Erythematosus Disease Activity Index-2,000 (SLEDAI-2000) and analyzed usingy Spearman's test. IFIT4, interferon-induced protein with tetratricopeptide repeats 4.

Spearman's correlation analysis was carried out to determine the relationship between IFIT4 expression and the clinical characteristics of SLE. We found that IFIT4 mRNA relative expression correlated with ANA titre in 108 SLE patients (*r *= 0.4783, *P *< 0.001; Figure 2a), and with anti-dsDNA autoantibody titre in 36 SLE patients (*r *= 0.3932, *P *< 0.05; Figure 2b), and with anti-Sm antibody titre in seven SLE patients (*r *= 0.9088, *P *< 0.01; Figure 2c). Moreover, SLE patients who were positive for anti-dsDNA autoantibodies had higher IFIT4 expression than did those who were negative (*P *= 0.0277; Figure 2d). The difference in IFIT4 expression between patients who were positive or negative for anti-Ro (anti-SSA; Figure 2e) or anti-aCL/β2-GP1 (Figure 2f) was not statistically significant (*P *> 0.05). Our findings suggest that higher IFIT4 expression is associated with a greater likelihood of having autoantibodies against ribonucleoproteins.

### Correlation between the expression of IFIT4 and clinical manifestation in SLE

In the SLE population as a whole, we found that SLE patients with hypocomplementaemia or leucopenia or/and thrombocytopenia usually had higher IFIT4 mRNA relative expression than did SLE patients without these disorders (*P *= 0.0121, Figure 2g; *P *= 0.0301, Figure 2h). However, IFIT4 expression in PBMCs from SLE patients with or without nephritis was not statistically different (*P *> 0.05; Figure 2i). We found no correlation between IFIT4 gene expression and scores of the SLEDAI-2K in 108 SLE patients (*r *= 0.0574, *P *> 0.05; Figure 2j).

### IFIT4 protein is expressed predominantly in immune tissues and cells, and exclusively localized in the cytoplasm

**Figure 3:**
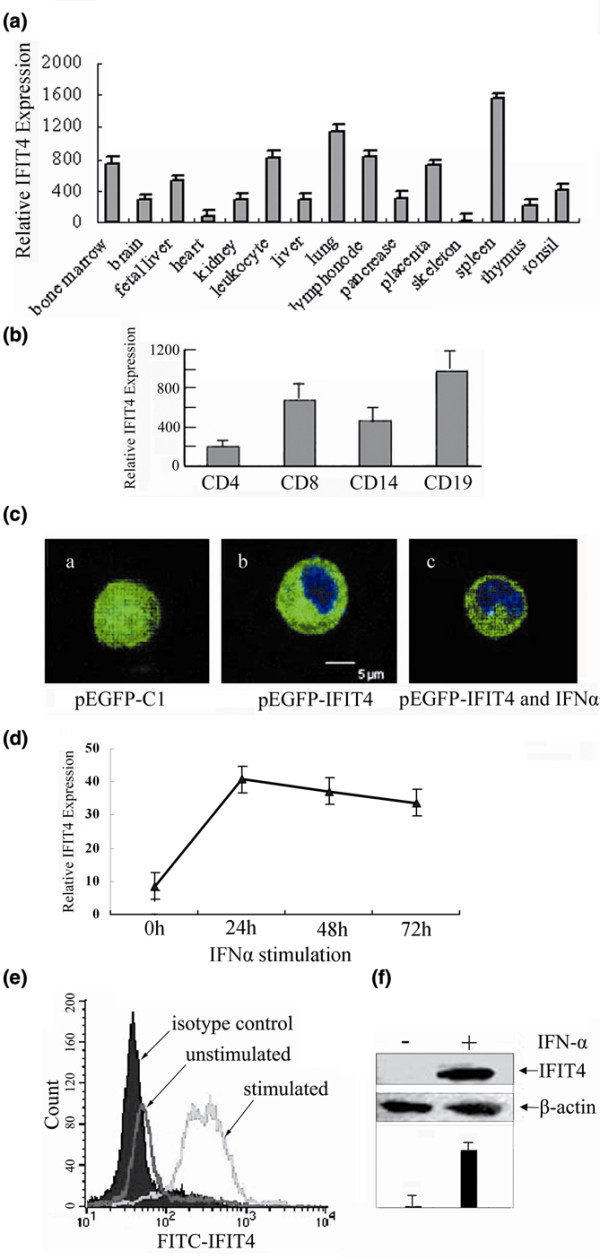
Distribution of IFIT4 in tissues and cells, and the effect of IFN-α on IFIT4 expression. Using real-time quantitative RT-PCR, IFIT4 mRNA relative expression was determined among **(a) **14 normal human tissues and **(b) **four kinds of immune cells. To determine the subcellular location of IFIT4 protein, THP-1 cells were transfected with **(c) **(subpanel a) pEGFP-C1 control or (subpanel b) pEGFP-IFIT4 plasmid. Forty-eight hours later, cells were stained with DAPI for nuclear staining and examined by confocal microscopy. The effect of IFN-α2a on the localization of IFIT4 protein was also observed. (c) (subpanel c) THP-1 cells transfected with pEGFP-IFIT4 were further stimulated with 3,000 μ/ml IFN-α2a for 72 hours. Blue colour shows the location of the nucleus, whereas green colour shows the sublocalization of green fluorescent protein alone or fused with IFIT4 protein. **(d) **To analyze the effect of IFN-α on the expression level of IFIT4, peripheral blood mononuclear cells (PBMCs) from healthy donors were treated with 3,000 μ/ml IFN-α2a for 24 hours, 48 hours or 72 hours, and then IFIT4 mRNA in the PBMCs was detected by real-time quatitative RT-PCR; all experiments were repeated three times with similar results. IFIT4 protein levels from normal PBMCs treated with IFN-α2a for 72 hours was examined by **(e) **flow cytometry with intracellular staining or **(f) **Western blotting with β-actin as a protein loading control. The experiments were performed three times and a set of representative histograms and data is presented. IFIT4, interferon-induced protein with tetratricopeptide repeats 4.

IFIT4 was examined using real-time quantitative RT-PCR and was found to be predominantly expressed in seven out of 14 normal human tissues, namely spleen, lung, leucocytes, lymph nodes, placenta, bone marrow and foetal liver (in order of the highest to the lowest), most of which belong to the immune system (*P *< 0.05; Figure 3a). Rather than being restricted to one immune cell, IFIT4 was expressed in many immune cells, including CD19^+^, CD8^+^, CD14^+ ^and CD4^+ ^cells (*P *< 0.05; Figure 3b).

To determine the the subcellular localization of IFIT4 protein, THP-1 cells were transfected with pEGFP-C1 or pEGFP-IFIT4 plasmid and examined by confocal microscopy (Figure 3c). In contrast to the pEGFP-C1 group, in which EGFP (enhanced green fluorescent protein) was dispersed throughout the cell (Figure 3c [subpanel a]), EGFP-IFIT4 fusion protein was specifically localized to the cytoplasm of THP-1 cells transfected with pEGFP-IFIT4 (Figure 3 [subpanel b]), and no translocation of IFIT4 into the nucleus was observed after 3 days of stimulation with IFN-α2a (Figure 3c [subpanel c]).

### Strong induction of IFIT4 by IFN-α2a *in vitro*

IFIT4 mRNA and protein in normal PBMCs was preferentially induced by IFN-α2a (3,000 units/ml), as observed using real-time quantitative RT-PCR, fluorescence-activated cell sorting (FACS), and Western blotting. The time course study revealed strong induction of IFIT4 mRNA by IFN-α2a as early as 24 hours after treatment (Figure 3d). FACS findings showed that IFIT4 protein was expressed in about 48.7% of normal PBMCs, with mean fluorescence intensity of 121.18. However, after exposure to IFN-α2a for 3 days, up to 99.47% PBMCs expressed IFIT4 protein with a mean fluorescence intensity of 327.97 (*P *< 0.05; Figure 3e). Upregulation of IFIT4 expression by IFN-α2a was confirmed by Western blotting (Figure 3f).

### Effect of IFIT4 on the morphological changes in the differentiation of monocytes into dendritic cells

**Figure 4:**
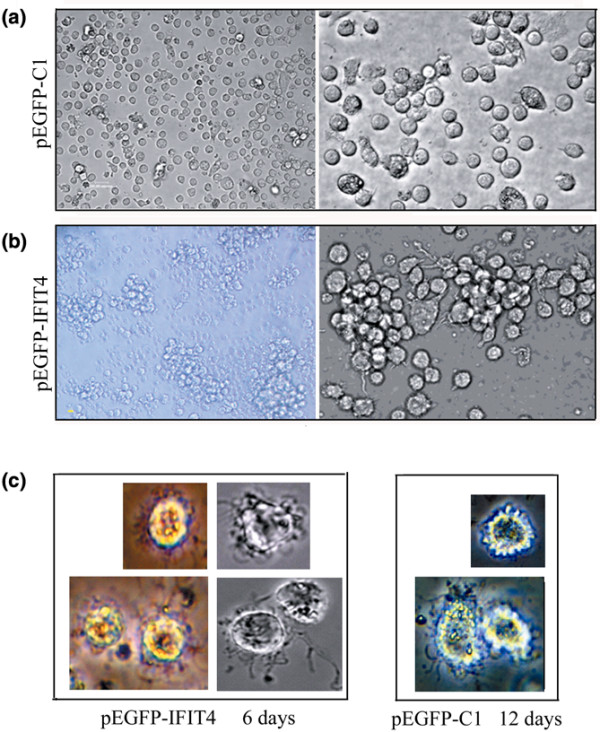
Morphological comparison of DCLCs primed with or without IFIT4 over-expression by inverted microscope. Cell morphology changes were observed to evaluate the effects of IFIT4 on dendritic cell (DC) differentiation upon treatment with granulocyte-macrophage colony-stimulating factor (GM-CSF; 50 ng/ml) or IL-4 (20 ng/ml). **(a) **THP-1 cells were transfected with pEGFP-C1; 36 hours later cells were further stimulated with GM-CSF/IL-4 for 86 hours (left lane: ×20; right lane: ×40). **(b) **THP-1 cells were transfected with pEGFP-IFIT4 fusion plasmid; 36 hours later cells were further stimulated with GM-CSF/IL-4 for 48 hours (left lane: ×20; right lane: ×40). **(c) **Effect of IFIT4 on morphological changes that occured in monocytes differentiation into mature and activated DC-like cells (DCLCs). THP-1 cells were transfected with pEGFP-IFIT4 (left lane) or pEGFP-C1 (right lane) plasmid; 36 hours later cells were stimulated with GM-CSF/IL-4 for 6 days (left lane) or 12 days (right lane) followed by lipopolysaccharide (LPS; 100 ng/ml) for another 2 days. All the cells above were observed with an inverted microscope. More than three fields of view (containing ≥100 cells/field) per sample were examined. IFIT4, interferon-induced protein with tetratricopeptide repeats 4.

Because it is usually difficult to transfect primary monocytes, we chose an appropriate monocytic cell line, namely THP-1, which has a suspended, rounded appearance and is widely used as a model for monocyte-macrophage differentiation [42-44,48,49]. We confirmed that THP-1 cells have the potential to differentiate into mature DCLCs with various characteristics of DC morphology (data not shown) after 12 days of treatment with GM-CSF/IL-4 followed by stimulation with LPS for another 2 days. An effect of IFIT4 on monocyte differentiation into DCs in terms of morphological changes was observed. Upon treatment with GM-CSF/IL-4 for 86 hours, no DC appearance was observed in THP-1 cells transfected with pEGFP-C1 vector (Figure 4a). Specifically, THP-1 cells remained dispersed and rounded (left lane: ×20) and were similar in size to intact THP-1 cells (right lane: ×40). In contrast, THP-1 cells primed with pEGFP-IFIT4 transfection partially acquired DC morphology after as little as 48 hours of GM-CSF/IL-4 treatment (Figure 4b). These IFIT4-primed DCLCs not only aggregated into clusters and became half adherent (left lane: ×20) but they also developed cytoplasmic protrusions or dendrites around the cell surface and increased in size (right lane: ×40). Moreover, THP-1 cells primed with pEGFP-IFIT4 transfection and stimulated with GM-CSF/IL-4 for 6 to 8 days plus 2 days of LPS exhibited the distinct mature and active morphology of DCs, manifesting as numerous processes and long veils, and dendrites (Figure 4c, left lane). However, 12 days of stimulation with GM-CSF/IL-4 was necessary for THP-1 cells primed with pEGFP-C1 transfection to differentiate into mature DCLCs (Figure 4c, right lane). These findings indicate that IFIT4-primed DCLCs adapted DC morphology sooner and had a greater resemblance to DCs than did THP-1 transfected with control plasmid upon the same GM-CSF/IL-4 treatment.

### Effects of IFIT4 on increasing the expression of costimulatory molecules in DCLCs

**Figure 5:**
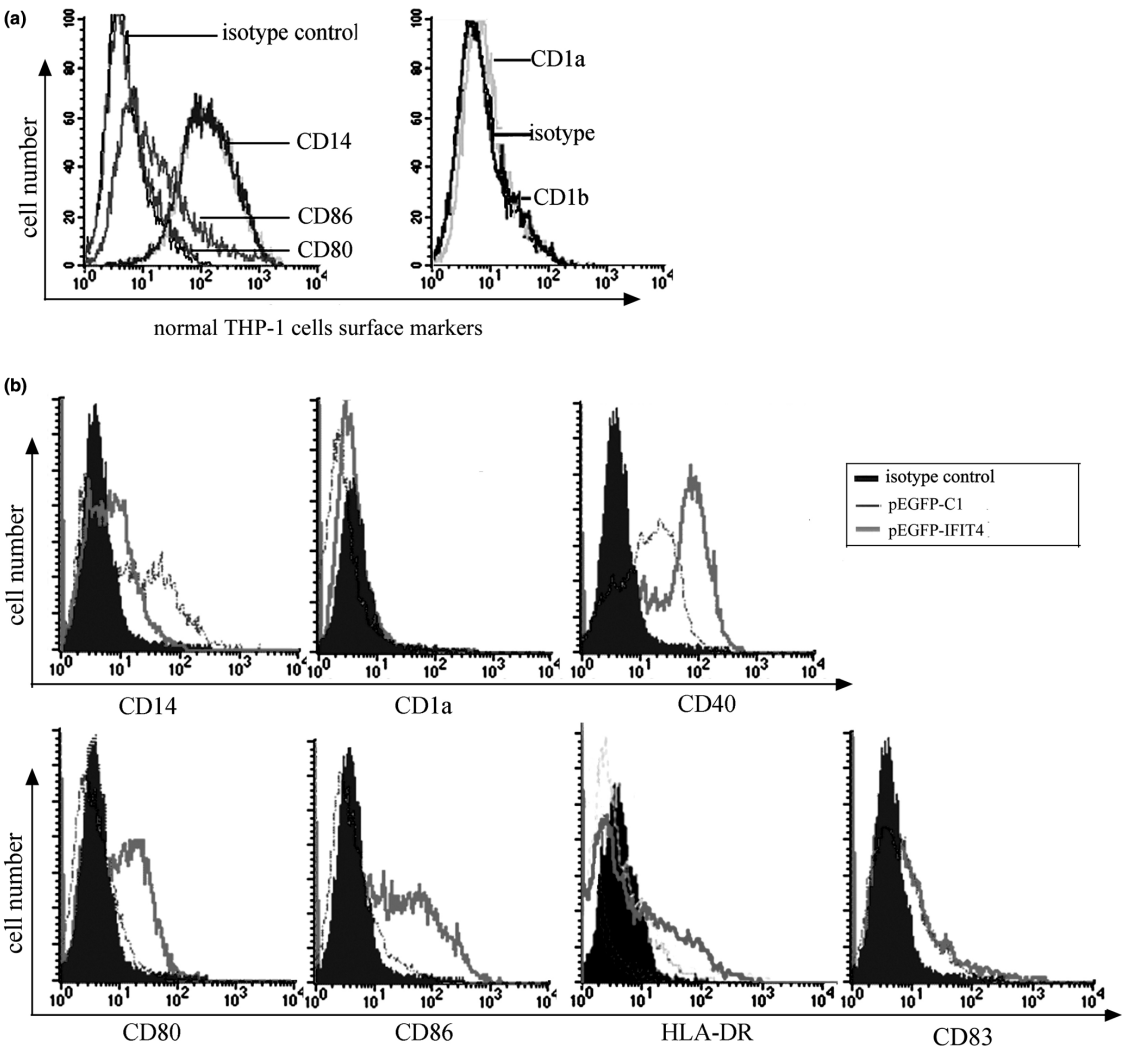
Comparison of the phenotypic profiles of DCLCs primed with or without IFIT4 over-expression. **(a) **Surface antigens of normal THP-1 cells were examined by flow cytometry. **(b) **To analyze the effect of IFIT4 on phenotypic changes of dendritic cell-like cells (DCLCs) during the process of differentiation, THP-1 cells were transfected with pEGFP-IFIT4 or pEGFP-C1; 36 hours later, cells were stimulated with granulocyte-macrophage colony-stimulating factor (GM-CSF; 50 ng/ml) and IL-4 (20 ng/ml) for 90 hours to generate DCLCs. These DCLCs primed with pEGFP-IFIT4 or pEGFP-C1 transfection were incubated with fluorochrome-conjugated monoclonal antibodies (mAbs) and the antigens of CD40, CD80, CD86, CD83, HLA-DR, CD14 and CD1a on the surface of those DCLCs were analyzed by flow cytometry. Appropriate fluorochrome or isotype control mAbs of each antibody species were used as negative controls. Shaded histograms represent isotype control antibodies. The thick line represents DCLCs primed with pEGFP-IFIT4 transfection, whereas the slender lines represent DCLCs primed with pEGFP-C1 transfection. All experiments were performed three times and a set of representative histograms was presented. IFIT4, interferon-induced protein with tetratricopeptide repeats 4.

We first assessed the expression background of related surface markers of THP-1 cells by FACS. We found that CD14 was expressed at high levels, whereas CD80, CD86, CD1a and CD1b were expressed at lower levels on the surface of intact THP-1 cells (Figure 5a), confirming that THP-1 cells were more consistent with a monocytic than a dendritic phenotype. To determine the effect of IFIT4 on DC differentiation in terms of cell phenotypic changes, THP-1 cells primed with pEGFP-C1 or pEGFP-IFIT4 transfection were treated with GM-CSF/IL-4 for 90 hours; the cell surface markers were analyzed by flow cytometry. We found that DCLCs primed with pEGFP-IFIT4 transfection expressed higher levels of CD40, CD80, CD86 and HLA-DR, but lower levels of CD14 than did those DCLCs primed with pEGFP-C1 transfection upon the same GM-CSF/IL-4 stimulation (*P *< 0.05; Figure 5b). There was no change in CD1a expression between the two groups (Figure 5b).

### Effect of IFIT4 in terms of enhancing the ability of DCLCs to present antigens to T cells

**Figure 6:**
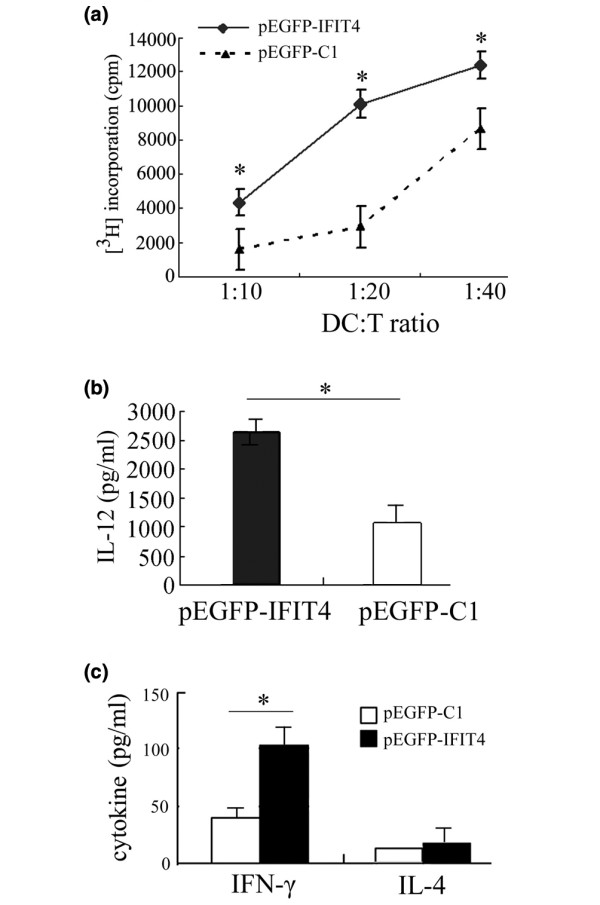
Functional analysis of IFIT4-primed DCLCs. Mature and activated dendritic cell-like cells (DCLCs) were generated from THP-1 cells transfected with pEGFP-IFIT4 or pEGFP-C1 and harvested after 6 days of culture with granulocyte-macrophage colony-stimulating factor (GM-CSF)/IL-4 plus 2 days of stimulation with lipopolysaccharide (LPS; 100 ng/ml). **(a) **Effect of IFIT4 on modulating the ability of DCLCs to present antigens to T cells was examined by allogeneic mixed leukocyte responses (MLRs). Allogeneic CD4^+ ^T cells were cultured for 3 days with γ-irradiated mature DCLCs primed with pEGFP-C1 or pEGFP-ITFIT4 transfection at different ratios of DCLCs to T cells (1:10, 1:20 and 1:40). The cells were harvested and the incorporated radioactivity was measured using a beta counter. Responses are reported as the mean of triplicate samples (counts/minute [cpm] ± standard deviation). **(b) **Effect of IFIT4 on IL-12 production by DCLCs. Mature and activated DCLCs were generated as described above. Supernatant from the DCLCs (1 × 10^5 ^cells/ml) primed with pEGFP-C1 or pEGFP-ITFIT4 transfection was analyzed for IL-12p70 by ELISA. **(c) **Analysis of the cytokine-production in T cells primed with co-stimulation by DCLCs. Mature and activated DCLCs primed with pEGFP-C1 or pEGFP-ITFIT4 transfection were generated as described above. Then these mature and activated DCLCs were γ-irradiated and cultured with T cells at a ratio of DCLCs to T cells of 1:10 for 6 days. Phorbol-12-myristate-13-acetate (PMA) (10 ng/ml) was added for another day. Finally, the culture medium was measured to assess IFN-γ and IL-4 produced by T cells by ELISA (T cells: 2 × 10^5 ^cells/ml; DCLC to T cell ratio = 1:10). DCLCs primed with pEGFP-IFIT4 (shaded histogram, test group) or pEGFP-C1 (open histogram, control group) were compared with each other. The results are expressed as the mean ± standard deviation. Data represent the mean of triplicate experiments. The asterisk indicates a highly significant difference between the test group and the control (*P *< 0.05). IFIT4, interferon-induced protein with tetratricopeptide repeats 4.

To examine further the effect of IFIT4 on modulating the ability of DCLCs to activate T-cell proliferation, an allogeneic MLR assay was conducted. Mature DCLCs primed with pEGFP-IFIT4 or pEGFP-C1 were generated with 6 days of GM-CSF/IL-4 stimulation and further treatment with 2 days of LPS, and then these DCLCs were cultured with allogeneic CD4^+ ^cells with different ratios of DCLC to T cells. As shown in Figure 6a, the extent of T-cell proliferation induced by DCLCs primed with pEGFP-IFIT4 transfection was significantly higher than those induced by DCLCs primed with pEGFP-C1, indicating that IFIT4-primed DCLCs had a greater ability to induce T-cell proliferation than controls (Figure 6a; *P *< 0.05).

### Effect of IFIT4 on IL-12 secretion by activated DCLCs

To determine the effect of IFIT4 on IL-12 secretion by activated DCLCs, THP-1 cells transfected with pEGFP-IFIT4 or pEGFP-C1 were stimulated with GM-CSF/IL-4 for 6 days and further treated with LPS for 2 days (1 × 10^5 ^cells/ml) to generate mature, activated DCLCs. We found that the mature and activated IFIT4-primed DCLCs produced more IL-12 than did DCLCs primed with pEGFP-C1 transfection (Figure 6b; *P *< 0.05).

### TH1 polarization by IFIT4-primed DCLCs

To address whether IFIT4 modulates the capacity of DCLCs to direct T-helper cell differentiation, mature and activated DCLCs were generated as described above. Then these mature DCLCs were γ-irradiated followed by incubation with T cells for 6 days. The ELISA findings revealed that T cells that were stimulated with IFIT4-primed mature DCLCs secreted more IFN-γ (about 2.4-fold) than did those stimulated with DCLCs primed with pEGFP-C1 transfection (*P *< 0.05; Figure 6c). We did not find that any of the DCLCs affected IL-4 secretion to a significant degree (Figure 6c; *P *> 0.05).

## Discussion

SLE is an autoimmune disease that is characterized by a break in tolerance to nuclear components and by multi-tissue damage. The pathogenesis of SLE has not been fully elucidated. Increased expression of a spectrum of IFIGs in SLE [3,30-32], the inherent responsiveness of these genes to Type I Interferon (IFN-I) [29,30], and the correlation between IFN-I or IFIGs and production of autoantibodies or disease severity [18,50] indicate a coordinated activation of the IFN-I pathway globally and a role for IFIGs in the pathogenesis of SLE [5,18]. In this study we found evidence that IFIT4, which was highly expressed in patients with SLE, is associated with the presence of autoantibodies and hypocomplementaemia as well as haematocytopenia. Most notably, IFIT4 might play a role in monocyte differentiation into DCs, and hence contribute to the mechanism by which autoantibodies become elevated and some of the clinical manifestations present in SLE.

We confirmed that IFIT4 mRNA and protein were significantly increased in PBMCs from SLE patients and positively correlated with the presence of ANA, anti-dsDNA and anti-Sm/RNP antinucleoprotein autoantibodies, which indicates that a role of IFIT4 in the pathogenesis of SLE might be directly or indirectly associated with the production of autoantibodies. SLE patients with higher expression of IFIT4 exhibited a greater prevalence of hypocomplementaemia, leucopenia and thrombocytopenia. The function of IFIT4 in antiproliferation [34] might be partially responsible for the haematocytopenia observed in patients with SLE, which requires further study in the future. There was no apparent relationship between IFIT4 expression and the presence of renal disease or SLEDAI. The clinical characteristics of IFIT4 are similar to those of another IFIG, namely Mx1, whose expression is also positively associated with autoantibodies against SM/RNP and dsDNA, but not with SLEDAI or the presence of renal disease [4]. IFN-α score [18] was derived from many IFIGs (PRKR, IFIT1 and IFI44) as measured by quantitative real-time PCR and represented the global activation of IFN-I pathway [3,18,29,30,51],. We presumed that, unlike the IFN-α score, IFIT4 or Mx1, as individual IFIG, was unable to be suggested to exclusively determine the global and comprehensive disease manifestation, severity and activity or even organ damage.

IFIT4 was found to be predominantly expressed in immune tissues and cells, indicating a potential role for IFIT4 in immune response. Both its extranuclear localization and its tetratricopeptide repeat motifs provide some clues that emphasize the role played by IFIT4 in the interaction between proteins via its tetratricopeptide repeat domain. That no translocation into nucleus took place after stimulation with IFN-α indicates that IFIT4 required other protein interaction partners to transport information into the nucleus. IFIT4 was markedly induced by IFN-α in normal PBMCs, indicating a role for IFIT4 as a downstream effector of IFN-α [28,29].

Both IFN-α [16,17,46] and IFIT4 were related to the presence of autoantibodies. IFN-α might indirectly contribute to the generation of autoantibodies by inducing monocytes to differentiate into DCs [2,41,52,53]. Our results showed that IFIT4 might be responsible for monocyte differentiation as a downstream effector of IFN-α. We found that the IFIT4-primed DCLCs, in which IFIT4 was over-expressed, adopted DC morphology sooner and exhibited a greater resemblance to DCs than did DCLCs that were primed with control pEGFP-C1 transfection, in terms of becoming half adherent and developing dendrites (Figure 4). This indicates that IFIT4 promoted the morphological changes that occurred during differentiation of monocytes into DCs. Furthermore, compared with control DLCLs, IFIT4-primed DCLCs exhibited a pattern of surface markers that was more consistent with DCs, including greater expression of the co-stimulatory molecules CD40, CD80, CD86, HLA-DR and CD83, along with more obvious downregulation of the monocytic marker CD14 (Figure 5). Moreover, activated IFIT4-primed DCLCs induced stronger MLR and produced more IL-12 than did control cells (Figure 6).

Collectively, these effects of IFIT4 on morphology, phenotype, antigen-presenting ability and IL-12 production of monocytes suggest that IFIT4 might play a role in promoting monocyte differentiation into DCs. This is because IFIT4-primed cells acquired more cytoplasmic protrusions or dendrites; more expression of the co-stimulatory molecules CD40, CD80, CD86, HLA-DR and CD83; lower expression of CD14; stronger antigen-presenting ability; and more IL-12 production. All of these are among the key characteristics of immature or mature DCs. Moreover, IFIT4-primed DCLCs stimulated greater IFN-γ secretion by T cells, which suggests that IFIT4 might direct DCs to modulate Th1 cell differentiation and favour the ability of DCs to skew the immune response toward Th1 development.

It was shown that monocytes from patients with SLE had severely altered phenotype and lineage flexibility, and might act as DCs [54,55]. For instance, they expressed significantly lower levels of CD14 [54] and were able to induce strong MLR [2], although HLA-DR levels were similar to [55] or lower than [54,56] those of controls. The effect of IFIT4 on monocyte differentiation into DCs was suggested to be partially responsible for this unbalanced function of monocytes and DCs in SLE [2]. The function of IFIT4 on differentiation and anti-proliferation [34] indicated that IFIT4 might act as a regulator to balance proliferation and differentiation.

How might IFN-α and IFIT4 contribute to the pathogenesis of SLE? Our hypothesis is that IFN-α and IFIT4 may act as 'adjuvant'-like factors [21] to convert an immune system that ignores self-antigens into an immune system that actively recognizes these antigens by promoting DC differentiation, maturation and activation [21,57]. The activated DCs may present ribonucleoprotein antigens in an immunogenic rather than tolerogenic manner, leading to the activation of autoreactive T cells that 'help' to drive autoantibody production in B cells [58].

## Conclusion

Our results indicate that IFIT4 might contribute to the pathogenesis of SLE by inducing monocytes to differentiate into DCs. This provides insight into the pathogenesis of SLE and might lead to novel treatments [25,59].

## Abbreviations

ANA = antinuclear antibody; anti-dsDNA = anti-double-stranded DNA antibody; DC = dendritic cell; DCLC = dendritic cell-like cell; ELISA = enzyme-linked immunosorbent assay; FACS = fluorescence-activated cell sorting; GAPDH = glyceraldehyde-3-phosphate dehydrogenase; GM-CSF = granulocyte-macrophage colony-stimulating factor; IFIG = interferon-inducible gene; IFIT4 = interferon-induced protein with tetratricopeptide repeats 4; IFN = interferon; IL = interleukin; LPS = lipopolysaccharide; MLR = mixed leukocyte response; PBMC = peripheral blood mononuclear cell; RT-PCR = reverse trancription polymerase chain reaction; SLE = systemic lupus erythematosus; SLEDAI = Systemic Lupus Erythematosus Disease Activity Index; Th1 = T-helper-1.

## Competing interests

The authors declare that they have no competing interests.

## Authors' contributions

NS, LW, XYH, CDB, SLC and YY G designed the study. XYH and NS conducted research. XYH, NS and LW analyzed data. XYH, NS and LW wrote the manuscript. All authors read and approved the final manuscript.

## References

[B1] Vakharia DD, Szebenyi SE, Gutterman JU, Rich SA (1996). Interferon-alpha-induced human lupus inclusions and p36 protein in cancer and AIDS. J Interferon Cytokine Res.

[B2] Blanco P, Palucka AK, Gill M, Pascual V, Banchereau J (2001). Induction of dendritic cell differentiation by IFN-alpha in systemic lupus erythematosus. Science.

[B3] Baechler EC, Batliwalla FM, Karypis G, Gaffney PM, Ortmann WA, Espe KJ, Shark KB, Grande WJ, Hughes KM, Kapur V, Gregersen PK, Behrens TW (2003). Interferon-inducible gene expression signature in peripheral blood cells of patients with severe lupus. Proc Natl Acad Sci USA.

[B4] Feng X, Wu H, Grossman JM, Hanvivadhanakul P, FitzGerald JD, Park GS, Dong X, Chen W, Kim MH, Weng HH, Furst DE, Gorn A, McMahon M, Taylor M, Brahn E, Hahn BH, Tsao BP (2006). Association of increased interferon-inducible gene expression with disease activity and lupus nephritis in patients with systemic lupus erythematosus. Arthritis Rheum.

[B5] Crow MK (2005). Interferon pathway activation in systemic lupus erythematosus. Curr Rheumatol Rep.

[B6] Santiago-Raber ML, Baccala R, Haraldsson KM, Choubey D, Stewart TA, Kono DH, Theofilopoulos AN (2003). Type-I interferon receptor deficiency reduces lupus-like disease in NZB mice. J Exp Med.

[B7] Ronnblom L, Eloranta ML, Alm GV (2006). The type I interferon system in systemic lupus erythematosus. Arthritis Rheum.

[B8] Selmi C, Lleo A, Zuin M, Podda M, Rossaro L, Gershwin ME (2006). Interferon alpha and its contribution to autoimmunity. Curr Opin Investig Drugs.

[B9] Kim T, Kanayama Y, Negoro N, Okamura M, Takeda T, Inoue T (1987). Serum levels of interferons in patients with systemic lupus erythematosus. Clin Exp Immunol.

[B10] Zhuang H, Kosboth M, Lee P, Rice A, Driscoll DJ, Zori R, Narain S, Lyons R, Satoh M, Sobel E, Reeves WH (2006). Lupus-like disease and high interferon levels corresponding to trisomy of the type I interferon cluster on chromosome 9p. Arthritis Rheum.

[B11] Dall'era MC, Cardarelli PM, Preston BT, Witte A, Davis JC (2005). Type I interferon correlates with serological and clinical manifestations of SLE. Ann Rheum Dis.

[B12] Bengtsson AA, Sturfelt G, Truedsson L, Blomberg J, Alm G, Vallin H, Ronnblom L (2000). Activation of type I interferon system in systemic lupus erythematosus correlates with disease activity but not with antiretroviral antibodies. Lupus.

[B13] Thibault DL, Utz PJ (2003). Interpreting interest in interferon-alpha. Arthritis Res Ther.

[B14] Borg FA, Isenberg DA (2007). Syndromes and complications of interferon therapy. Curr Opin Rheumatol.

[B15] Munoz-Rodriguez FJ, Andreu Solsona V, Tricas Leris JM, Vilaseca Bellsola J (2002). Interferon alpha-2b induced lupus erythematosus in a patient with chronic hepatitis C infection [in Spanish]. Rev Clin Esp.

[B16] Ioannou Y, Isenberg DA (2000). Current evidence for the induction of autoimmune rheumatic manifestations by cytokine therapy. Arthritis Rheum.

[B17] Ronnblom LE, Alm GV, Oberg KE (1991). Autoimmunity after alpha-interferon therapy for malignant carcinoid tumors. Ann Intern Med.

[B18] Kirou KA, Lee C, George S, Louca K, Peterson MG, Crow MK (2005). Activation of the interferon-alpha pathway identifies a subgroup of systemic lupus erythematosus patients with distinct serologic features and active disease. Arthritis Rheum.

[B19] Then Bergh F, Dayyani F, Ziegler-Heitbrock L (2004). Impact of type-I-interferon on monocyte subsets and their differentiation to dendritic cells. An *in vivo* and *ex vivo* study in multiple sclerosis patients treated with interferon-beta. J Neuroimmunol.

[B20] Zang YC, Skinner SM, Robinson RR, Li S, Rivera VM, Hutton GJ, Zhang JZ (2004). Regulation of differentiation and functional properties of monocytes and monocyte-derived dendritic cells by interferon beta in multiple sclerosis. Mult Scler.

[B21] Santini SM, Lapenta C, Logozzi M, Parlato S, Spada M, Di Pucchio T, Belardelli F (2000). Type I interferon as a powerful adjuvant for monocyte-derived dendritic cell development and activity *in vitro* and in Hu-PBL-SCID mice. J Exp Med.

[B22] Gabriele L, Borghi P, Rozera C, Sestili P, Andreotti M, Guarini A, Montefusco E, Foa R, Belardelli F (2004). IFN-alpha promotes the rapid differentiation of monocytes from patients with chronic myeloid leukemia into activated dendritic cells tuned to undergo full maturation after LPS treatment. Blood.

[B23] Della Bella S, Nicola S, Riva A, Biasin M, Clerici M, Villa ML (2004). Functional repertoire of dendritic cells generated in granulocyte macrophage-colony stimulating factor and interferon-alpha. J Leukoc Biol.

[B24] Luft T, Pang KC, Thomas E, Hertzog P, Hart DN, Trapani J, Cebon J (1998). Type I IFNs enhance the terminal differentiation of dendritic cells. J Immunol.

[B25] Lee PY, Reeves WH (2006). Type I interferon as a target of treatment in SLE. Endocr Metab Immune Disord Drug Targets.

[B26] Paquette RL, Hsu NC, Kiertscher SM, Park AN, Tran L, Roth MD, Glaspy JA (1998). Interferon-alpha and granulocyte-macrophage colony-stimulating factor differentiate peripheral blood monocytes into potent antigen-presenting cells. J Leukoc Biol.

[B27] Hardin JA (2003). Directing autoimmunity to nucleoprotein particles: the impact of dendritic cells and interferon alpha in lupus. J Exp Med.

[B28] Baechler EC, Gregersen PK, Behrens TW (2004). The emerging role of interferon in human systemic lupus erythematosus. Curr Opin Immunol.

[B29] Kirou KA, Lee C, George S, Louca K, Papagiannis IG, Peterson MG, Ly N, Woodward RN, Fry KE, Lau AY, Prentice JG, Wohlgemuth JG, Crow MK (2004). Coordinate overexpression of interferon-alpha-induced genes in systemic lupus erythematosus. Arthritis Rheum.

[B30] Crow MK, Kirou KA, Wohlgemuth J (2003). Microarray analysis of interferon-regulated genes in SLE. Autoimmunity.

[B31] Bennett L, Palucka AK, Arce E, Cantrell V, Borvak J, Banchereau J, Pascual V (2003). Interferon and granulopoiesis signatures in systemic lupus erythematosus blood. J Exp Med.

[B32] Han GM, Chen SL, Shen N, Ye S, Bao CD, Gu YY (2003). Analysis of gene expression profiles in human systemic lupus erythematosus using oligonucleotide microarray. Genes Immun.

[B33] Dauffy J, Mouchiroud G, Bourette RP (2006). The interferon-inducible gene, Ifi204, is transcriptionally activated in response to M-CSF, and its expression favors macrophage differentiation in myeloid progenitor cells. J Leukoc Biol.

[B34] Xiao S, Li D, Zhu HQ, Song MG, Pan XR, Jia PM, Peng LL, Dou AX, Chen GQ, Chen SJ, Chen Z, Tong JH (2006). RIG-G as a key mediator of the antiproliferative activity of interferon-related pathways through enhancing p21 and p27 proteins. Proc Natl Acad Sci USA.

[B35] de Veer MJ, Sim H, Whisstock JC, Devenish RJ, Ralph SJ (1998). IFI60/ISG60/IFIT4, a new member of the human IFI54/IFIT2 family of interferon-stimulated genes. Genomics.

[B36] Huang X, Yang N, Ou X, Li D, Wang Z, Xie Q, Chen Y, Lin H, Yin G, Wen F (2008). Sequential activation of protein kinase C delta and JNK is required for interferon-alpha-induced expression of IFIT4. Cell Signal.

[B37] Yu M, Tong JH, Mao M, Kan LX, Liu MM, Sun YW, Fu G, Jing YK, Yu L, Lepaslier D, Lanotte M, Wang ZY, Chen Z, Waxman S, Wang YX, Tan JZ, Chen SJ (1997). Cloning of a gene (RIG-G) associated with retinoic acid-induced differentiation of acute promyelocytic leukemia cells and representing a new member of a family of interferon-stimulated genes. Proc Natl Acad Sci USA.

[B38] Palucka AK, Banchereau J, Blanco P, Pascual V (2002). The interplay of dendritic cell subsets in systemic lupus erythematosus. Immunol Cell Biol.

[B39] Zhao KW, Li X, Zhao Q, Huang Y, Li D, Peng ZG, Shen WZ, Zhao J, Zhou Q, Chen Z, Sims PJ, Wiedmer T, Chen GQ (2004). Protein kinase Cdelta mediates retinoic acid and phorbol myristate acetate-induced phospholipid scramblase 1 gene expression: its role in leukemic cell differentiation. Blood.

[B40] Yoshida Y, Sakaguchi H, Ito Y, Okuda M, Suzuki H (2003). Evaluation of the skin sensitization potential of chemicals using expression of co-stimulatory molecules, CD54 and CD86, on the naive THP-1 cell line. Toxicol In Vitro.

[B41] Dauer M, Schad K, Junkmann J, Bauer C, Herten J, Kiefl R, Schnurr M, Endres S, Eigler A (2006). IFN-alpha promotes definitive maturation of dendritic cells generated by short-term culture of monocytes with GM-CSF and IL-4. J Leukoc Biol.

[B42] Puig-Kroger A, Serrano-Gomez D, Caparros E, Dominguez-Soto A, Relloso M, Colmenares M, Martinez-Munoz L, Longo N, Sanchez-Sanchez N, Rincon M, Rivas L, Sanchez-Mateos P, Fernandez-Ruiz E, Corbi AL (2004). Regulated expression of the pathogen receptor dendritic cell-specific intercellular adhesion molecule 3 (ICAM-3)-grabbing nonintegrin in THP-1 human leukemic cells, monocytes, and macrophages. J Biol Chem.

[B43] Luo YP, Li YG, Cai DC, Ren H (2002). Study on induction of dendritic cells from myeloid leukemia cell lines and their antitumor immune function [in Chinese]. Zhongguo Shi Yan Xue Ye Xue Za Zhi.

[B44] Brach MA, Riedel D, Herrmann F (1990). Induction of monocytic differentiation and modulation of the expression of c-fos, c-fms and c-myc protooncogenes in human monoblasts by cytokines and phorbolester. Virchows Arch B Cell Pathol Incl Mol Pathol.

[B45] Chen XQ, Yang J, Hu SP, Nie HX, Mao GY, Chen HB (2006). Increased expression of CD86 and reduced production of IL-12 and IL-10 by monocyte-derived dendritic cells from allergic asthmatics and their effects on Th1- and Th2-type cytokine balance. Respiration.

[B46] Hua J, Kirou K, Lee C, Crow MK (2006). Functional assay of type I interferon in systemic lupus erythematosus plasma and association with anti-RNA binding protein autoantibodies. Arthritis Rheum.

[B47] Chan RW, Tam LS, Li EK, Lai FM, Chow KM, Lai KB, Li PK, Szeto CC (2003). Inflammatory cytokine gene expression in the urinary sediment of patients with lupus nephritis. Arthritis Rheum.

[B48] Ishiguro A, Spirin KS, Shiohara M, Tobler A, Gombart AF, Israel MA, Norton JD, Koeffler HP (1996). Id2 expression increases with differentiation of human myeloid cells. Blood.

[B49] Charrad RS, Gadhoum Z, Qi J, Glachant A, Allouche M, Jasmin C, Chomienne C, Smadja-Joffe F (2002). Effects of anti-CD44 monoclonal antibodies on differentiation and apoptosis of human myeloid leukemia cell lines. Blood.

[B50] Zhuang H, Narain S, Sobel E, Lee PY, Nacionales DC, Kelly KM, Richards HB, Segal M, Stewart C, Satoh M, Reeves WH (2005). Association of anti-nucleoprotein autoantibodies with upregulation of Type I interferon-inducible gene transcripts and dendritic cell maturation in systemic lupus erythematosus. Clin Immunol.

[B51] Crow MK, Kirou KA (2004). Interferon-alpha in systemic lupus erythematosus. Curr Opin Rheumatol.

[B52] Paquette RL, Hsu N, Said J, Mohammed M, Rao NP, Shih G, Schiller G, Sawyers C, Glaspy JA (2002). Interferon-alpha induces dendritic cell differentiation of CML mononuclear cells *in vitro *and *in vivo*. Leukemia.

[B53] Tosi D, Valenti R, Cova A, Sovena G, Huber V, Pilla L, Arienti F, Belardelli F, Parmiani G, Rivoltini L (2004). Role of cross-talk between IFN-alpha-induced monocyte-derived dendritic cells and NK cells in priming CD8+ T cell responses against human tumor antigens. J Immunol.

[B54] Steinbach F, Henke F, Krause B, Thiele B, Burmester GR, Hiepe F (2000). Monocytes from systemic lupus erythematous patients are severely altered in phenotype and lineage flexibility. Ann Rheum Dis.

[B55] Koller M, Zwolfer B, Steiner G, Smolen JS, Scheinecker C (2004). Phenotypic and functional deficiencies of monocyte-derived dendritic cells in systemic lupus erythematosus (SLE) patients. Int Immunol.

[B56] Nagai H, Sztein MB, Steeg PS, Hooks JJ, Oppenheim JJ, Steinberg AD (1984). Diminished peripheral blood monocyte DR antigen expression in systemic lupus erythematosus. Clin Exp Rheumatol.

[B57] Banchereau J, Pascual V (2006). Type I interferon in systemic lupus erythematosus and other autoimmune diseases. Immunity.

[B58] Bell DA, Morrison B, Bygaart P Vanden (1990). Immunogenic DNA-related factors. Nucleosomes spontaneously released from normal murine lymphoid cells stimulate proliferation and immunoglobulin synthesis of normal mouse lymphocytes. J Clin Invest.

[B59] Crow MK (2003). Interferon-alpha: a new target for therapy in systemic lupus erythematosus?. Arthritis Rheum.

